# Prediction of Response to Induction Chemotherapy Plus Concurrent Chemoradiotherapy for Nasopharyngeal Carcinoma Based on MRI Radiomics and Delta Radiomics: A Two-Center Retrospective Study

**DOI:** 10.3389/fonc.2022.824509

**Published:** 2022-04-22

**Authors:** Yuzhen Xi, Xiuhong Ge, Haiming Ji, Luoyu Wang, Shaofeng Duan, Haonan Chen, Mengze Wang, Hongjie Hu, Feng Jiang, Zhongxiang Ding

**Affiliations:** ^1^Department of Radiology, Key Laboratory of Clinical Cancer Pharmacology and Toxicology Research of Zhejiang Province, Affiliated Hangzhou First People’s Hospital, Cancer Center, Zhejiang University School of Medicine, Hangzhou, China; ^2^Department of Radiology, 903rd Hospital of PLA, Hangzhou, China; ^3^Department of Radiology, Liangzhu Hospital, Hangzhou, China; ^4^GE Healthcare, Precision Health Institution, Shanghai, China; ^5^Zhejiang Chinese Medical University, Hangzhou, China; ^6^Department of Radiology, Sir Run Run Shaw Hospital Affiliated to Medical College Zhejiang University, Hangzhou, China; ^7^Department of Head and Neck Radiotherapy, Zhejiang Cancer Hospital/Zhejiang Province Key Laboratory of Radiation Oncology, Hangzhou, China

**Keywords:** nasopharyngeal carcinoma, magnetic resonance imaging, radiomics, induction chemotherapy, concurrent chemoradiotherapy

## Abstract

**Objective:**

We aimed to establish an MRI radiomics model and a Delta radiomics model to predict tumor retraction after induction chemotherapy (IC) combined with concurrent chemoradiotherapy (CCRT) for primary nasopharyngeal carcinoma (NPC) in non-endemic areas and to validate its efficacy.

**Methods:**

A total of 272 patients (155 in the training set, 66 in the internal validation set, and 51 in the external validation set) with biopsy pathologically confirmed primary NPC who were screened for pretreatment MRI were retrospectively collected. The NPC tumor was delineated as a region of interest in the two sequenced images of MRI before treatment and after IC, followed by radiomics feature extraction. With the use of maximum relevance minimum redundancy (mRMR) and least absolute shrinkage and selection operator (LASSO) algorithms, logistic regression was performed to establish pretreatment MRI radiomics and pre- and post-IC Delta radiomics models. The optimal Youden’s index was taken; the receiver operating characteristic (ROC) curve, calibration curve, and decision curve were drawn to evaluate the predictive efficacy of different models.

**Results:**

Seven optimal feature subsets were selected from the pretreatment MRI radiomics model, and twelve optimal subsets were selected from the Delta radiomics model. The area under the ROC curve, accuracy, sensitivity, specificity, negative predictive value (NPV), and positive predictive value (PPV) of the MRI radiomics model were 0.865, 0.827, 0.837, 0.813, 0.776, and 0.865, respectively; the corresponding indicators of the Delta radiomics model were 0.941, 0.883, 0.793, 0.968, 0.833, and 0.958, respectively.

**Conclusion:**

The pretreatment MRI radiomics model and pre- and post-IC Delta radiomics models could predict the IC-CCRT response of NPC in non-epidemic areas.

## Introduction

Nasopharyngeal carcinoma (NPC) is a malignant tumor originating from the nasopharyngeal mucosal epithelium, which is sensitive to radiotherapy (1). Global Cancer Statistics of 2020 indicated 133,354 new cases of NPC and 80,008 deaths worldwide (2, 3). Meanwhile, the incidence and mortality of NPC in China are higher than the global average estimate. Radiotherapy is the primary treatment for NPC, and concurrent chemoradiotherapy (CCRT) can improve the radiotherapy effect by shrinking the tumor, increasing radiosensitivity, and reducing the radiation dose (4). The 2019 National Comprehensive Cancer Network Clinical Practice Guidelines have recommended induction chemotherapy (IC) combined with CCRT (IC-CCRT) as a class 2A modality for the treatment of advanced NPC (5). In recent years, there has been increasing evidence that IC-CCRT or radiotherapy has clinical value in improving progression-free survival (PFS) and relapse-free survival (RFS) of NPC patients (6). IC-CCRT is also effective in the treatment of non-endemic areas of NPC (7). Although NPC treatment has improved with the advancement of chemoradiotherapy strategies, the 5-year survival rate of some patients with advanced disease is about 60%–85%, and the therapeutic efficacy remains unsatisfactory (8, 9).

The development of treatment options and the evaluation of prognosis for NPC mainly depend on the tumor node metastasis (TNM) stage. However, anatomy-based TNM staging only reflects the tumor shape and invasion into surrounding structures and ignores the internal characteristics of the tumor with the same stage. Hence, despite receiving similar treatment regimens, about 20% of patients show unsatisfactory results due to individual differences and tumor heterogeneity (10). Radiochemotherapy resistance remains one of the main causes of poor prognosis and treatment failure in NPC (11), while residual mass is an independent factor for poor prognosis (12, 13). Treatment of residual disease is associated with better survival outcomes compared to the treatment of recurrent tumors (14). As the tumor shrinks during treatment, adjacent normal brain tissue, skull base bone, and other tissues will fall into the high-dose irradiated tumor areas, increasing the risk of radiation-related injury (15). Therefore, it is necessary to reveal the heterogeneity of tumors as early as possible, facilitating to predict tumor shrinkage in individualized and precise treatment and prognosis of NPC patients.

Radiomics has become a popular method to study tumor heterogeneity in recent years. It can describe tumor heterogeneity and other features by mining the high-dimensional quantitative characteristics of standard medical images (CT, MRI, PET, etc.), providing clinical and high-throughput quantitative information and more personalized treatment options (16). Radiomics characteristics are usually defined in two ways (17), including single-time point radiology and Delta radiomics. Single-time point radiology is mostly used before or during treatment to establish a genomics characteristics model for diagnosis (18, 19), tumor risk stratification (20–23), and prognosis prediction (24, 25), associated with higher powers compared with the TNM staging system. Delta radiomics uses radiological features during or after treatment to provide a wealth of information to identify and quantify treatment-induced changes to guide clinical decisions. It may be more suitable for assessing tumor treatment efficacy (26). Some studies have shown that Delta radiomics-based models yield higher powers than single-time-point-based models (27–29).

Existing radiomics guidelines have recommended the use of multicenter data to ensure the generalizability of the findings (30, 31). However, for many studies, there are very little external validation data. In addition, Delta radiomics studies in predicting adverse events in head and neck squamous cell carcinoma are mostly based on CT imaging (32, 33). The aim of this study was to construct a pretreatment MRI radiomics model and Delta radiomics models before and after IC and to explore their application values in dynamically predicting chemoradiotherapy efficacy for the treatment of NPC in non-epidemic regions.

## Materials and Methods

### Patients

**Figure 1:**
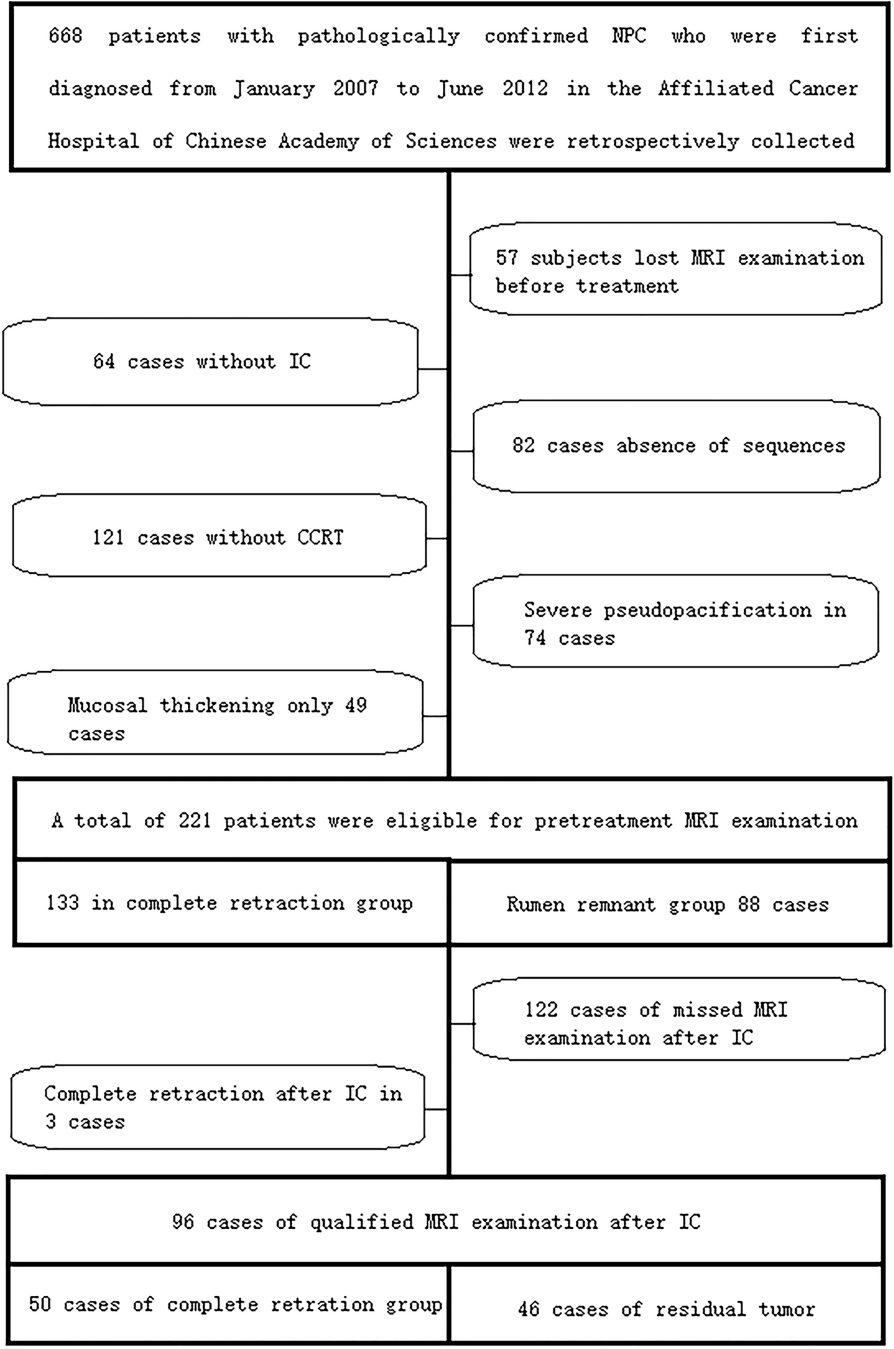
The case screening process in the training set and internal validation set. A total of 221 qualified cases were screened before the final treatment, including 96 qualified cases after IC. IC, induction chemotherapy.

The study was approved by the local scientific research ethics committee, and informed consent was waived due to its retrospective nature. The study process was in accordance with the Declaration of Helsinki. The information of 668 included patients with pathologically confirmed NPCat the Cancer Hospital of the University of Chinese Academy of Sciences (Zhejiang Cancer Hospital) was collected between January 2007 and June 2012. Then screening was performed according to the following conditions: 1) NPC diagnosis was pathologically confirmed; 2) patients were treated with IC-CCRT; 3) MRI examination was performed within 2 weeks before and after CCRT treatment; 3) fat-suppressed (FS) T2-weighted imaging (T2WI) and FS contrast-enhanced T2-weighted imaging (CE-T1WI) images were available. The patient selection process is shown in **Figure 1**. Then the data of patients with primary NPC in Sir Run Run Shaw Hospital, Zhejiang University, during the same period were re-screened as external validation data. A total of 51 qualified cases were identified before treatment, including 33 qualified cases after IC.

According to the Response Evaluation Criteria in Solid Tumors (RECIST Version 1.1), NPC patients with response to IC-CCRT were assigned to the complete tumor retraction group. The total retraction group was defined as no evidence of residual disease on reexamination of MRI, that is, complete response (CR). The residual group was defined as residual disease on reexamination of MRI images after IC-CCRT, including partial response (PR), stable disease (SD), or progressive disease (PD).

### MRI Acquisition

MRI images of all patients were obtained using two different MRI scanners (Siemens Magnetom Symphony 1.5T and Siemens SKyra 3.0T, Munich, Germany). Axial FS T2WI images were initially obtained, followed by axial FS CE-T1WI imaging after gadolinium-based contrast agent administration at 0.01 mmol/kg. The acquisition protocol for neck MRI was slightly different but mainly consisted of the following parameters: 1) axial FS T2WI, repetition time/echo time (TR/TE) 6,360 ms/95 ms, 90° flip angle, 256 × 168 matrix, slice thickness 4.68 mm, slice spacing 4.68 mm; and 2) axial FS CE-T1WI TR/TE 450 ms/8.8 ms, 90° flip angle, 256 × 168 matrix, slice thickness 4.68 mm, slice spacing 4.68 mm.

### MRI Preprocessing and Image Segmentation

The nasopharyngeal MRI examinations before treatment, after IC, and after CCRT of each case were searched from the hospital PACS, and the DICOM format images of FS T2WI and FS CE-T1WI sequences were exported. DICOM format images were imported using ITK-SNAP (www.itksnap.org, version 3.8.0) software. We first selected axial FS CE-T1WI images, in order to improve the accuracy of lesion delineation, and axial FS T2WI images were imported in the “Add Another DICOM Series” option. Two attending radiologists with 7 and 8 years of experience in head and neck radiological diagnosis manually segmented the primary NPC tumor bodies layer by layer. The same approach was used when tumor bodies were delineated on the axial FS T2WI images. Finally, the original images and segmented images were stored according to the format requirements. The same method was applied for tumor segmentation in 96 patients after IC.

### Radiomics Feature Extraction and Delta Radiomics Feature Calculation

The study used the software of Artificial Intelligence Kit (AK) V3.4.0.R issued by GE Company (Chicago, IL, USA). This software has been registered, approved, and applied to medical radiomics research (34–36). First, the unsegmented original data were imported into AK software, the original data were resampled, the resolution was adjusted to 1 mm × 1 mm × 1 mm, the layer thickness was 1 mm, the image gray level was uniformly adjusted to 0–255, and then the region of interest (ROI) image was imported. First Order, Shape, GLCM, GLDM, GLSZM, GLRLM, and NGTDM were used for feature selection, and LoG, Wavelet, and LBP were used for filter selection. Radiomics features were extracted from MRI images of 221 patients before treatment and 96 patients after IC.

For Delta radiology profile estimation, the change in each radiology profile was calculated by the following equation:


Delta Feature value=(Feature value2−Feature value1)

Here, Feature value2 represents the post-IC MRI value, and Feature value1 represents the pretreatment MRI value.

### Feature Selection, Model Establishment, and Statistical Analysis

All statistical analyses were performed using R statistical software (version 4.0.3). The intraclass correlation coefficient (ICC) was used to evaluate inter- and intra-observer agreement. Two attending radiologists randomly selected 40 patients and segmented the tumor once and twice again to calculate observer stability for each feature. Radiological features with ICC greater than 0.75 were defined as stable features. In order to ensure that the images from different devices were comparable, the data were normalized before feature selection, that is, Z-score transformation, and the calculation formula was as follows:


$$\text{z}=\frac{\bar{\text{x}}-\text{μ}}{\text{σ}/\sqrt{\text{n}}}$$

Here, μ is the mean, σ is the standard deviation, and n is the sample size.

**Figure 2:**
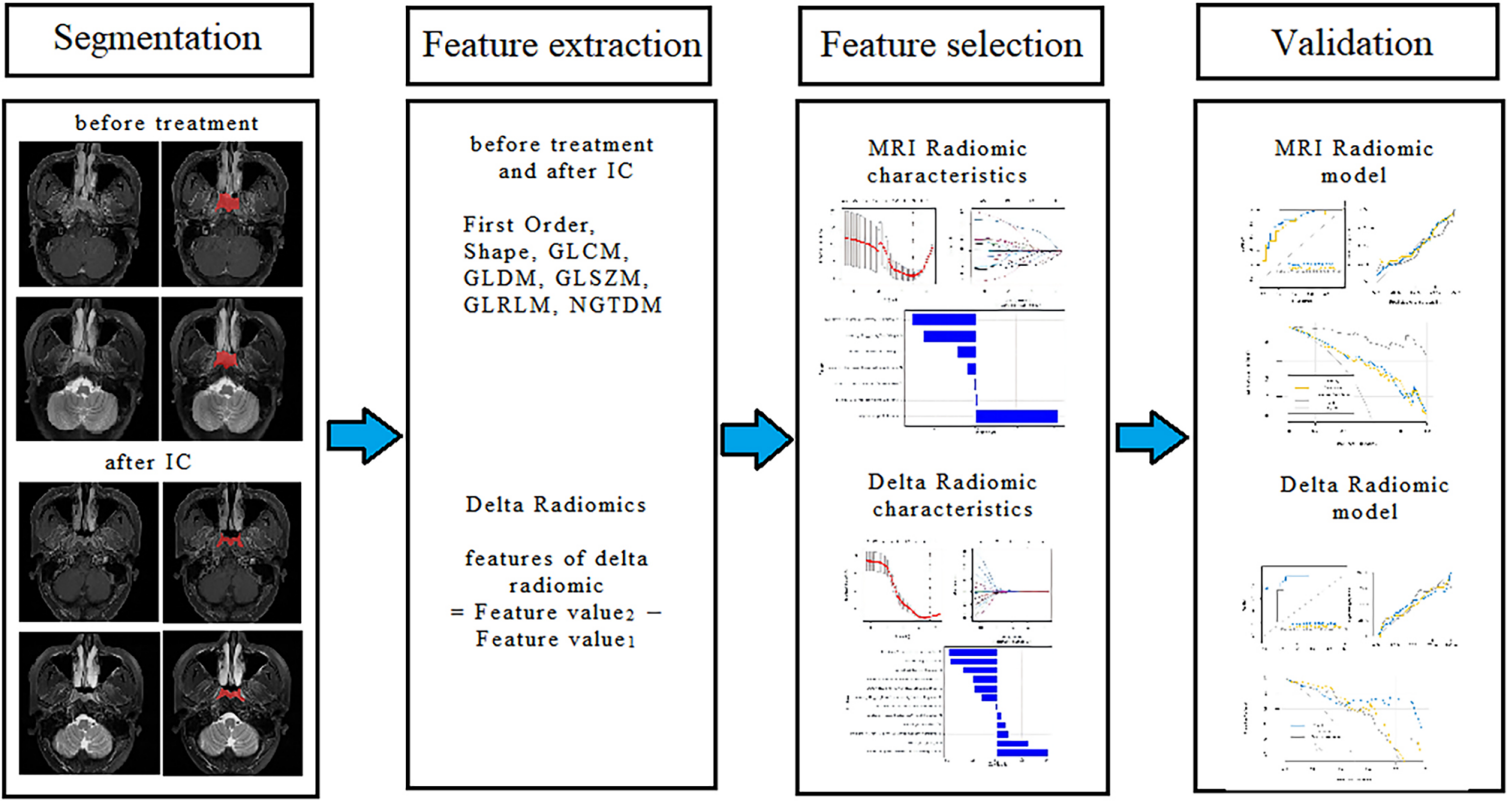
Flow diagram of radiomics.

The subjects were divided into the training group and the validation cohort in a ratio of 7:3. Then, maximum relevance minimum redundancy (mRMR) and least absolute shrinkage and selection operator (LASSO) were used to select features. We first used mRMR to retain 20 features that were maximally correlated with tags and least redundant with each other and then used LASSO to select the optimal subset of features for model construction. In order to avoid overfitting, we used 10 times of cross-validation to select the adjustment parameter λ. According to the screened characteristics and corresponding coefficients, a logistic regression model of FS T2WI combined with FS CE-T1WI was established, and the radiomics signature (Radscore) was obtained. The area under the receiver operating characteristic (ROC) curve (AUC), diagnostic accuracy, sensitivity, specificity, positive predictive value (PPV), and negative predictive value (NPV) of the training set and the internal validation set were calculated. ROC analysis was used to evaluate the diagnostic efficacy of the model. Calibration analysis was used to evaluate the goodness-of-fit of the model, and the decision curve was used to evaluate the clinical value of the model. The radiomics flowchart is shown in **Figure 2**.

## Results

### Analysis of Radiomics Results

**Figure 3:**
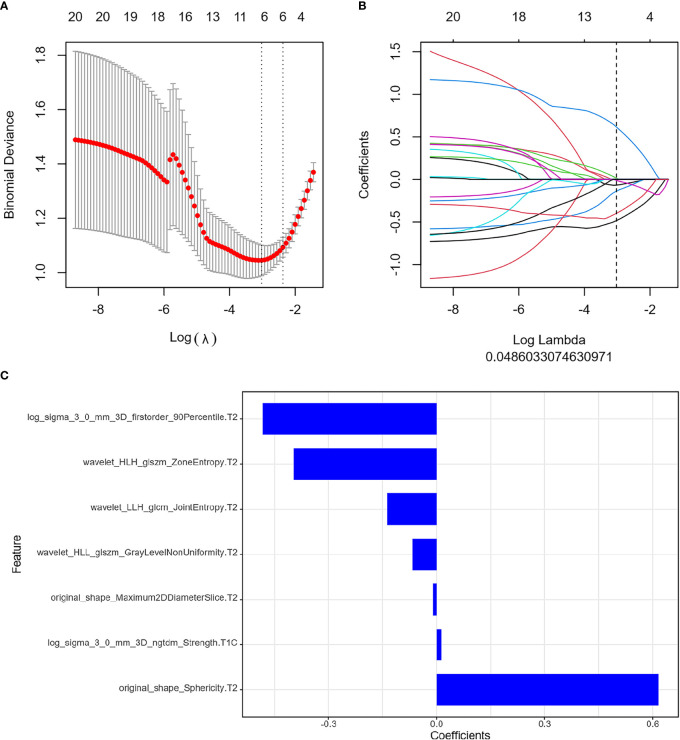
Feature extraction before treatment. **(A)** Uses ten-fold cross-validation and minimum criteria to select the adjustment parameter (λ). **(B)** LASSO coefficients of 20 texture features, drawing vertical lines at the selected optimal value in λ sequence. **(C)** Screens seven features. LASSO, least absolute shrinkage and selection operator.

**Figure 4:**
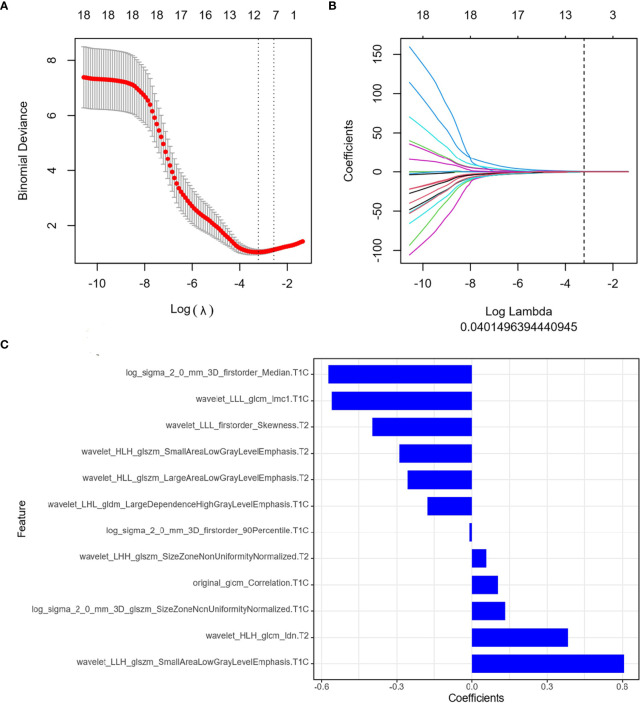
Feature extraction of Delta radiomics. **(A)** Uses ten-fold cross-validation and minimum criteria to select the adjustment parameter (λ). **(B)** LASSO coefficients of 20 texture features, drawing vertical lines at the selected optimal value in λ sequence. **(C)** Screens twelve features. LASSO, least absolute shrinkage and selection operator.

The ICC results were all greater than 0.75, and finally, the segmentation results of the senior doctor were selected. First, we selected 20 features that were most relevant to the tags and least redundant with each other among more than 2,000 features of the two sequences of FS T2WI and FS CE-T1WI by the mRMR method (**Figures 3A, B**; **4A, B**). Then LASSO was performed to select the optimal feature subset for constructing the model. After 10 cross-validations, six and twelve optimal subsets were finally retained (**Figures 3C**, **4C**). The Radscore was then calculated for each patient, and the results were detailed in the Appendix.

### Power Analysis of the Model to Predict the Efficacy of Induction Chemotherapy Combined With Concurrent Chemoradiotherapy

**Figure 5:**
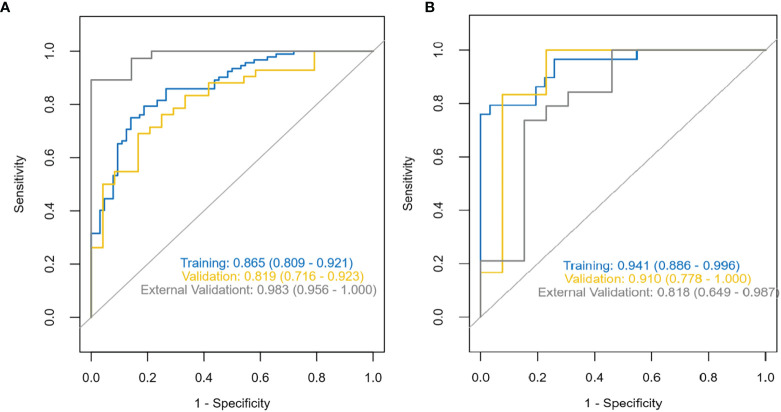
ROC characteristic curves and corresponding AUC values for the pretreatment MRI radiomics model **(A)** and the Delta radiomics model **(B)**. ROC, receiver operating characteristic; AUC, area under the receiver operating characteristic curve.

**Figure 6:**
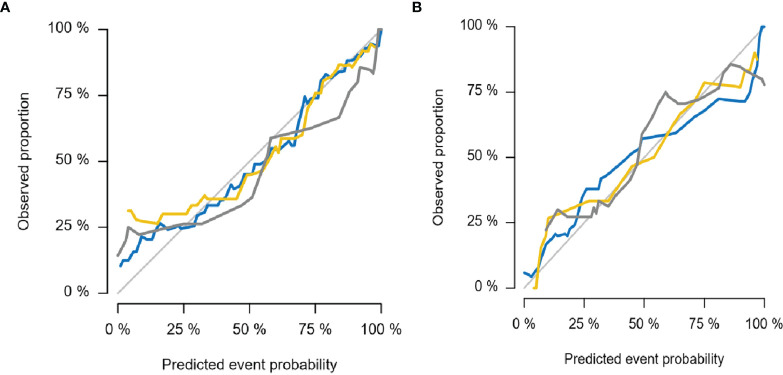
Calibration curves for MRI radiomics model **(A)** and Delta radiomics model **(B)**. The thin gray line represents the ideal reference line, the blue line represents the training set, the yellow line represents the internal validation set, and the thick gray line represents the external validation set. In this reference line, regardless of the training set, internal validation set, or external validation set, the predicted probability matches the observed proportion, indicating that both models have good performance in judging the tumor retraction of NPC primary tumors after IC-CCRT treatment. NPC, nasopharyngeal carcinoma; IC-CCRT, induction chemotherapy combined with concurrent chemoradiotherapy.

**Figure 7:**
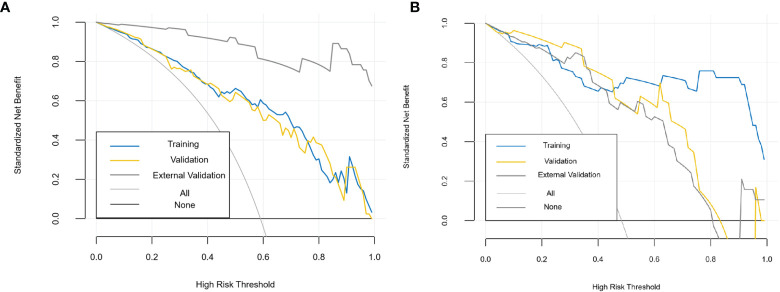
Decision curves of MRI radiomics model **(A)** and Delta radiomics model **(B)**. The x-axis represents the probability of response to IC-CCRT, ranging from 0% to 100%; the y-axis measures the net benefit; the blue, yellow, and coarse gray lines represent nomograms of different groups. IC-CCRT, induction chemotherapy combined with concurrent chemoradiotherapy.

The diagnostic effects of the pretreatment MRI radiomics model and Delta radiomics model in predicting the efficacy of IC-CCRT for NPC are summarized in **Table 1**. The AUC values (**Figure 5**), calibration curves (**Figure 6**), and decision curves (**Figure 7**) of both models showed good performance in the training set, internal validation set, and external validation set.

**Table 1:** The diagnostic effects of the pretreatment MRI radiomics model and Delta radiomics model in predicting the efficacy of IC-CCRT for NPC.

		ROC	Accuracy	Sensitivity	Specificity	NPV	PPV
MRI radiomics	Training set	0.865	0.827	0.837	0.812	0.776	0.865
	Validation set	0.819	0.788	0.833	0.708	0.708	0.833
	External validation	0.983	0.784	0.703	1.000	0.560	1.000
Delta radiomics	Training set	0.941	0.883	0.793	0.968	0.833	0.958
	Validation set	0.910	0.880	1.000	0.769	1.000	0.800
	External validation	0.818	0.781	0.737	0.846	0.688	0.875

ROC, receiver operating characteristic; NPV, negative predictive value; PPV, positive predictive value.

## Discussion

We selected the seven most relevant radiological features (six from FS T2WI images and one from FS CE-T1WI images) from the pretreatment MRI radiomics model of NPC and twelve most relevant radiological features (seven from FS CE-T1WI images and five from FS T2WI images) from the Delta radiomics model. The clinical predictive values of the two models after chemoradiotherapy for NPC were also analyzed. The results showed that the two constructed models had a high diagnostic performance for NPC, and there were some differences between the two models.

In this study, we selected two MRI sequences, FS T2WI and FS CE-T1WI, for the following three reasons. First, MRI was the most effective and commonly used method for the diagnosis and staging of NPC (37). MRI-based radiomics could better define tumor biology and had immense potential to support oncological decisions (38, 39). Second, these two sequences were commonly obtained during conventional MRI scans and might be more universal. Furthermore, FS sequence and enhanced examination could enhance the image contrast between the tumor and surrounding tissues, and lesion delineation was more accurate. The radiomics characteristics extracted from the combined images were reported to have better predictive performance compared with a single sequence.

In recent years, radiomics has been increasingly used in the diagnosis, tumor stratification, and prognosis prediction of NPC. For example, our previous study showed that the NPC radiomics model based on ^18^F-FDG PET/MRI images had immense value in the staging evaluation of primary NPC (36). The NPC could be divided into subtypes with different survival patterns based on the radiological characteristics of MRI (40). The early metastasis of NPC could be predicted based on Epstein–Barr virus (EBV), clinical data, and MRI nomogram. Radiomics could predict the PFS of NPC (41), and some radiomics characteristics could identify the patients who needed adaptive radiotherapy (42) and those who would benefit the most from adjuvant therapy or IC (43, 44). A combined model constructed based on EBV, clinical data, and radiological characteristics can predict NPC progression (45) and distant metastasis. However, few of these studies have external validation data, and there have been no reports on Delta radiomics of tumor retraction after IC-CCRT for NPC tumors. Our constructed MRI radiomics model at a single time point before treatment and Delta radiomics before and after IC could predict the efficacy of IC-CCRT for NPC for early prediction during treatment.

The optimal features of the pretreatment MRI radiomics model and Delta radiomics model focused on first-order characteristics, log and texture features after wavelet filtering, and morphological features of the original data. First-order statistics describe the distribution of voxel intensity in the image area defined by the mask through commonly used and basic metrics. About 90th percentile of first-order features and the changes in median and skewness are highly correlated with tumor retraction. The median value represents the gray intensity in the ROI, the asymmetry of the distribution of bias measurement, and the correlation value of the mean value. Sphericity characteristics of morphology in the MRI radiomics model, the roundness of the tumor area relative to the sphere morphology, were significantly correlated with tumor retraction. Our study showed that GLSM, GLCM, and NGTDM were also highly correlated with tumor retraction. GLSZM-ZE represented the uncertainty/randomness of the size of the measurement area and the gray level; the greater the value, the higher the heterogeneity. GLSZM-GLZE represented the distribution of the lower gray level area, and the higher value, the lower the gray value and the proportion of the size area in the image. GLCM describes the texture by studying the spatial correlation of the gray level. Energy represents the uncertainty and randomness of the image. The value of GLCM Joint Entropy represents the complexity of the co-occurrence matrix. In addition to first-order features in the Delta radiomics model, and SALGLE and LALGLE features in GLCM-IMC1, GLSZM is highly correlated with tumor retraction, which can quantify the complexity of texture. SALGLE measures the proportion of smaller sizes with lower gray values in regional joint distribution images. LALGLE measures the proportion of larger sizes with lower gray values in regional joint distribution images. These characteristics were most pronounced after chemoradiation for NPC in this study. Therefore, both the pretreatment MRI radiomics model and Delta radiomics model could predict tumor retraction after IC-CCRT.

However, this study had several limitations. First, this study was a retrospective analysis, and there was inevitable selectivity bias. Second, after IC, the cycle of CCRT was not fixed. We plan to collect more relevant data at a later stage and do more in-depth studies in the future. Third, because the data after treatment of IC were not uniform and the performance of the two models could not be compared, there was a general problem of poor refolding in radiomics. We are going to collect more cases to train the stability of the Delta radiomics model and increase its general applicability.

## Conclusion

Pretreatment MRI radiomics at a single time point and Delta radiomics before and after IC could predict tumor retraction after IC-CCRT for the treatment of NPC in non-endemic areas. This study provided a quantitative basis for early intervention and timely optimization of treatment options.

## Data Availability Statement

The original contributions presented in the study are included in the article/**Supplementary Material**. Further inquiries can be directed to the corresponding authors.

## Ethics Statement

The studies involving human participants were reviewed and approved by the Cancer Hospital of the University of Chinese Academy of Sciences (Zhejiang Cancer Hospital), Affiliated Hangzhou First People’s Hospital and Sir Run Run Shaw Hospital affifiliated to Medical College Zhejiang University. The patients/participants provided their written informed consent to participate in this study.

## Author Contributions

ZD designed and planned the study. XG, MW, HC, YX, HH, and FJ collected the clinical and image data. YX and HJ performed ROI segmentation. YX, SD, and LW analyzed the data. YX participated in drafting and writing the manuscript. All authors contributed to this study and approved the submitted version.

## Funding

This study was supported by grants from the National Natural Science Foundation of China (No. 81871337), Natural Science Foundation of Zhejiang Province (Y22H185692, LGF18H160018, and LY16H180007), Zhejiang Provincial Health Science and Technology Project (No. 2018KY304, No. 2020RC092, and No. 2021RC108), and Hangzhou Medical and Health Science and Technology Project (No. A20200507).

## Conflict of Interest

Author SD was employed by GE Healthcare.

The remaining authors declare that the research was conducted in the absence of any commercial or financial relationships that could be construed as a potential conflict of interest.

## Publisher’s Note

All claims expressed in this article are solely those of the authors and do not necessarily represent those of their affiliated organizations, or those of the publisher, the editors and the reviewers. Any product that may be evaluated in this article, or claim that may be made by its manufacturer, is not guaranteed or endorsed by the publisher.
