# Supplementary material for: Prediction of Response to Induction Chemotherapy Plus Concurrent Chemoradiotherapy for Nasopharyngeal Carcinoma Based on MRI Radiomics and Delta Radiomics: A Two-Center Retrospective Study
Source: Front Oncol. 2022 Apr 22;12:824509. doi: 10.3389/fonc.2022.824509 (PMC9074388; doi:10.3389/fonc.2022.824509)
Supplement: Supplementary file 1 [file DataSheet_1.docx]

Pretreatment Model: Radscore = (Intercept) 0.431983387725516 + waveletHLLglszmGrayLevelNonUniformity. T2-0.0671241444752755 + originalshapeSphericity. T2 * 0.615503681434656 + logsigma30 mm3Dfirstorder90Percentile. T2-0.483932617446627 + logsigma30 mm3DngtdmStrength. T1C0.0130826549432948 + waveletLLHglcmJointEntropy. T2-0.137900039259979 + originalshapeMaximum2DDiameterSlice. T2-0.0101363459726235 + waveletHLHglszmZoneEntropy. T2-0.398263559906045;

Delta radiomics model: Radscore = 0.410693976460653*wavelet_LLL_glcm_Imc1.T1C+0.356353751388776*log_sigma_2_0_mm_3D_firstorder_90Percentile.T1C+-0.0844901164455713*wavelet_LHH_gldm_SmallDependenceLowGrayLevelEmphasis.T1C+-0.0964600205930352*wavelet_LLH_glcm_MaximumProbability.T1C+-0.11776035397141*wavelet_LLH_glszm_SmallAreaLowGrayLevelEmphasis.T1C+-0.203946774608004*wavelet_LHL_ngtdm_Busyness.T2+-0.214778686437421*wavelet_LHL_ngtdm_Contrast.T2+-0.22068977666525*original_shape_SurfaceVolumeRatio.T1C+-0.285328938965854*wavelet_LHH_glszm_LargeAreaHighGrayLevelEmphasis.T1C+-0.30375058021162*wavelet_LLH_glszm_LowGrayLevelZoneEmphasis.T1C+-0.326376677430103*original_glcm_Correlation.T1C+-0.557595069298789*log_sigma_2_0_mm_3D_firstorder_Skewness.T1C+-0.100568992809806
